# The viral load monitoring cascade in a resource-limited setting: A prospective multicentre cohort study after introduction of routine viral load monitoring in rural Lesotho

**DOI:** 10.1371/journal.pone.0220337

**Published:** 2019-08-28

**Authors:** Tracy Renee Glass, Lipontso Motaboli, Bienvenu Nsakala, Malebanye Lerotholi, Fiona Vanobberghen, Alain Amstutz, Thabo Ishmael Lejone, Josephine Muhairwe, Thomas Klimkait, Niklaus Daniel Labhardt

**Affiliations:** 1 Swiss Tropical & Public Health Institute, Basel, Switzerland; 2 University of Basel, Basel, Switzerland; 3 SolidarMed, Swiss Organisation for Health in Africa, Butha-Buthe, Lesotho; 4 Ministry of Health of Lesotho, Maseru, Lesotho; 5 University Hospital Basel, Division of Infectious Diseases and Hospital Epidemiology, Basel, Switzerland; 6 Molecular Virology, Department of Biomedicine, University of Basel, Basel, Switzerland; University of Pittsburgh Centre for Vaccine Research, UNITED STATES

## Abstract

**Introduction:**

For HIV-positive individuals on antiretroviral therapy (ART), the World Health Organization (WHO) recommends routine viral load (VL) monitoring. We report on the cascade of care in individuals with unsuppressed VL after introduction of routine VL monitoring in a district in Lesotho.

**Materials and methods:**

In Butha-Buthe district 12 clinics (11 rural, 1 hospital) send samples for VL testing to the district laboratory. We included data from patients aged ≥15 years from Dec 1, 2015 to November 1, 2018. As per WHO guidelines VL <1000 copies/mL are considered suppressed, those ≥1000copies/mL unsuppressed. Patients with unsuppressed VL receive adherence counseling and follow-up VL within 8–12 weeks. Two consecutively unsuppressed VLs should trigger switch to second-line ART. For analysis of the VL monitoring cascade we defined care to be “according to guidelines” if patients with unsuppressed VL received a follow-up VL within <180 days and follow-up VL was either re-suppressed, or again unsuppressed and the individual was switched to second-line within 90 days.

**Results:**

For 9,949 individuals 24,948 VL tests were available. The majority were female (73%), median age 41 years (interquartile range 33–52), and 58% seen at rural clinics. Overall, 25% (260/1028) of individuals were managed according to guidelines: 40% (410/1028) had a follow-up VL within 180 days of their initial unsuppressed VL and 25% (260/1028) of those either re-suppressed or switched to second-line within 90 days. Female patients were more likely to have a follow-up VL done, (p = 0.015). In rural clinics rates of two consecutively unsuppressed VLs were higher than in the hospital (64% vs. 44%, p<0.001), and rural clinics were less likely to switch these patients to second-line (35% vs. 66%, p<0001).

**Conclusions:**

Our data show that in a real-life setting availability of routine VL monitoring may not be exploited to its potential. A lack of timely follow-up after a first unsuppressed VL and reluctance to switch patients with confirmed virological failure, reduce the benefit of VL monitoring, i.e. in the rural clinics. Future studies will have to assess models of care which ensure that VL results are met with an action and make use of scalable innovative approaches.

## Introduction

Since 2013, the World Health Organization (WHO) recommends viral load (VL) as the preferred monitoring strategy in persons living with HIV, who are taking antiretroviral therapy (ART)[1]. This recommendation was driven by evidence that cheaper alternatives, such as clinical monitoring and CD4 cell counts, are unreliable proxies to viral suppression[2]. As a result of the new WHO recommendations, resource-limited countries and international donors have been investing in scaling up VL testing since 2013[3]. Most low- and middle-income countries have now integrated VL monitoring in their national guidelines, but only few countries provide reliable access to the technically demanding and expensive procedure of regular VL monitoring [3,4].

In case of virological failure (≥1000 copies/mL), the WHO recommends enhanced adherence counselling and a follow-up VL after 3 months. A second VL ≥ 1000 copies/mL despite good adherence is defined as confirmed virological failure and should trigger the switch to a second-line regimen[1]. While from a clinical perspective there is no doubt that VL is the method of choice to monitor the treatment success of ART, from a public health perspective its impact in resource-limited settings still remains to be proven[5]. To have an impact, VL testing must become part of a package of good care with reliably rapid turn-around times of VL results, correct interpretation by staff, feedback to the patient and appropriate subsequent action[6,7]. Several studies on VL monitoring in resource-limited settings indicate, however, large gaps along the VL cascade of care: in particular, delays in follow-up VL or in switching to second-line ART after confirmed virological failure hamper the effectiveness of VL monitoring [8,9].

In Lesotho, a pilot study conducted prior to nation-wide scale-up of VL monitoring showed that among adult patients taking first-line ART, who had a first-time unsuppressed viral load, 90% attended enhanced adherence counseling and 84% had follow-up VL within 3–6 months. Out of those with confirmed virological failure, only 73% were switched to second-line while the remaining continued on a failing regimen[10]. Routine data may, however, look again different. Here we present first programmatic data after roll-out of routine VL monitoring in one district in Lesotho, presenting the VL cascade in a real-life setting.

## Methods

### Study design

Routine viral load monitoring was introduced in the district of Butha-Buthe, Lesotho, in December 2015. A prospective open cohort study was started to include all HIV-positive individuals on ART who have received a VL test. This study includes all adult patients (age≥15 years) in the cohort with at least one recorded VL result between December 1, 2015 and March 1, 2018. Follow-up for these participants continued until November 1, 2018, to allow 180 days follow-up for individuals with unsuppressed VL.

### Setting

The district of Butha-Buthe is characterized by mostly rural areas with an estimated population of 130,000, mainly subsistence farmers, mine workers, and construction or domestic labourers who work in neighbouring South Africa. The main town of Butha-Buthe has approximately 25,000 inhabitants with the remaining population living in villages scattered over a mountainous area. According to the recent household-based national survey from 2017 the adult HIV prevalence in the district is 17.8% [11].

Roll-out of routine VL monitoring in November 2015 started in Butha-Buthe district hospital’s ART clinic, the only urban site. The 11 rural clinics of the district began sending patients’ blood samples to the hospital laboratory for routine VL monitoring in June 2016. The schedule for routine VL monitoring follows current National Guidelines of Lesotho with a VL done after the first 6 months on ART and, if suppressed, yearly thereafter. In case of unsuppressed VL (≥1000 copies/mL), the guidelines recommend enhanced adherence counselling with a follow-up VL after 8–12 weeks. Sustained unsuppressed VL despite good adherence should trigger switch to a second-line regimen. In the case of resuppression or a VL log-drop of >0.5, the individual is maintained on first-line ART [12].

HIV care in Lesotho is nurse-based. At roll-out of VL monitoring, health professionals at all clinics were retrained on the National Guidelines, including the algorithm for VL monitoring and the criteria for switch to second-line ART.

### Data collection

Venous blood samples are collected in EDTA tubes at the clinics, transported the same day to the laboratory at the district hospital where they are centrifuged. Depending on work load, plasma samples are thereafter directly processed or stored in a -80°C freezer until the sample can be processed. Upon sample arrival at the laboratory, a data clerk enters the patient-related information from the national VL request form into the Laboratory Information System (LIS) developed by the Ministry of Health and utilized by all laboratories in Lesotho. Plasma samples are processed on a COBAS AmpliPrep/COBAS TaqMan according to the manufacturer’s instructions and with regular external auditing and quality controls. VL test results feed automatically into the LIS. On a weekly basis, trained laboratory technicians export the VL determinations into a password-protected database. The database contains demographic, treatment- and laboratory information. Each new laboratory record from the LIS is reviewed by data clerks, and health facilities are contacted for missing or inconsistent data.

### Outcomes

#### The viral load cascade

The main objective of this paper is to describe the VL care cascade. In line with current WHO recommendations we defined a VL <1000 copies/mL as “suppressed”, a VL ≥1000 copies/mL as “unsuppressed”. “Virological failure” is defined by WHO to be two consecutive VL measurements ≥1000 copies/mL despite good adherence.

To evaluate the VL cascade in individuals with a first unsuppressed VL, two steps were evaluated: 1) obtaining a follow-up VL and 2) actions taken based on the follow-up VL result.

We defined care to be “according to guidelines” if (1) a patient received a follow-up VL within < 180 days of his/her initial unsuppressed VL, and (2) the follow-up VL was either re-suppressed (<1000 copies/mL), or the follow-up VL was again ≥ 1000 copies/mL and the individual was switched to a 2^nd^-line regimen within 90 days or already on a second-line regimen.

#### The turn-around time of VL results

We further assessed variables reflecting the timeliness of VL monitoring: time from blood draw to registration at the hospital laboratory (registration time) and time from registration of the blood sample to testing (processing time). Turn-around time for a sample was defined as the time of blood-draw to the VL test being performed (registration time plus processing time). Turn-around times greater than 28 days were defined as “delayed”.

### Statistical methods

Results are summarized as frequencies and percentages for categorical variables and medians and interquartile ranges (IQR) for continuous variables. Comparisons were made using chi-square tests for categorical variables and Wilcoxon rank sum test of medians for continuous variables. For all tests we used two-sided p-values. All analyses were done using Stata v14 (Stata Corp, College Station, TX).

### Ethics statement

This prospective open cohort study has been approved by the National Health and Research Ethics Committee of the Ministry of Health of Lesotho (ID 134–2016). As documentation of VL results in the database is part of good routine care the Ethics Committee gave waiver of patient consent for the anonymous descriptive analysis of the routinely collected data.

## Results

### Patient characteristics

Over the 2.5-year study period, a total of 24,948 VL tests were done from 9,949 adults with a median of 3 VL tests (interquartile range [IQR] 2–3) per person. Table 1 displays patient characteristics.

**Table 1:** Patient characteristics.

	Individuals
N individuals	9949
Rural health facility	5806 (58%)
Age in years, median (IQR^$^)	41 (33–52)
Sex, female	7221 (73%)
First recorded CD4 count, cells/mm3, median (IQR)	248 (124–387) [n = 8912]
On first line ART	9828 (99.9%) [n = 9842]*
Years since ART initiation, median (IQR)	3 (1–6) [n = 9786]*

^$^IQR = interquartile range

*Due to missing data

### The viral load cascade

**Fig 1:**
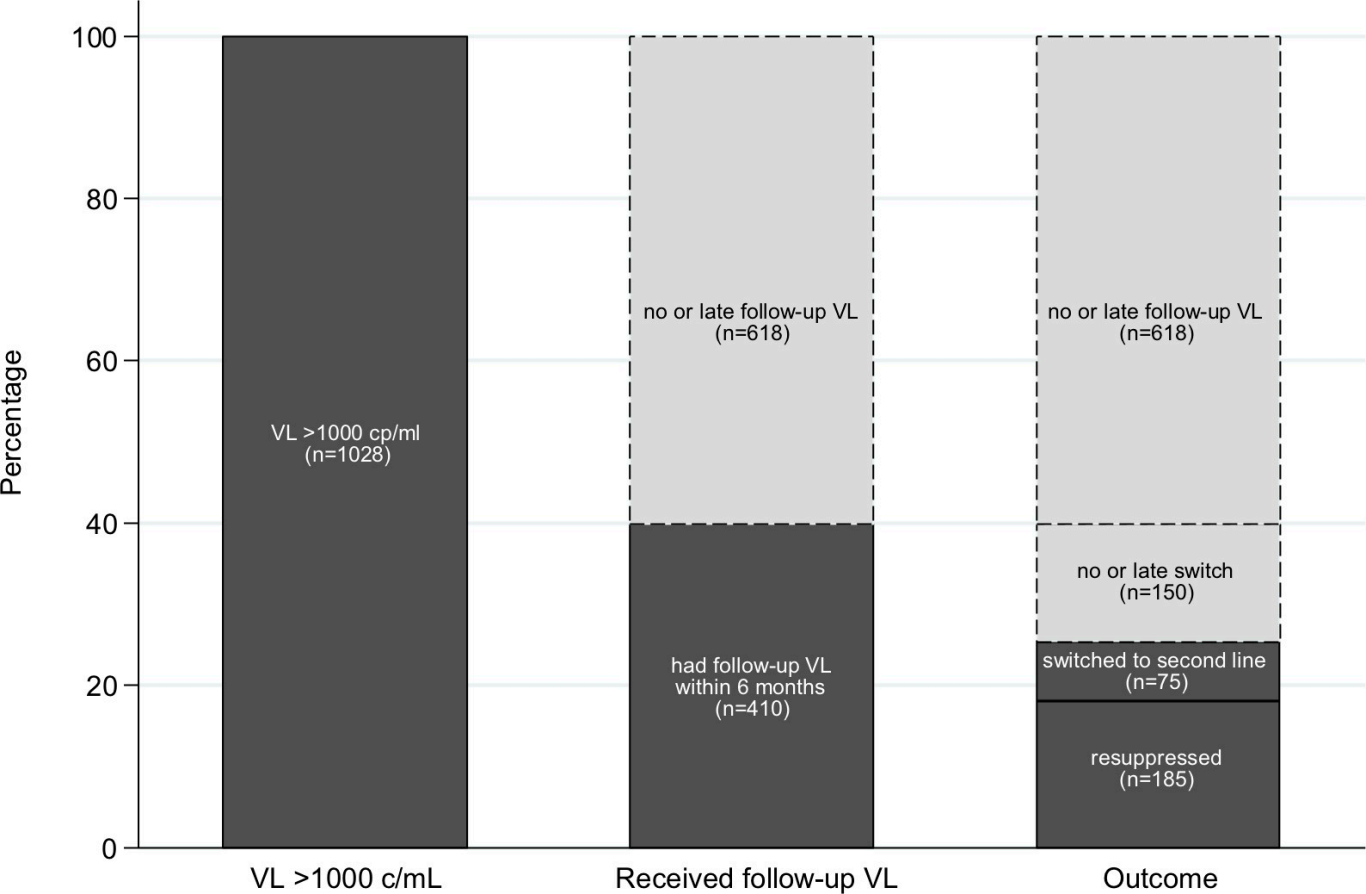
Viral load cascade.

During the study-period 1028 (11%) had at least one VL≥1000 copies/ml. Fig 1 displays the care cascade in these patients. Overall, only 25% (260/1028) of individuals were managed according to guidelines: 40% (410/1028) had a follow-up VL within 180 days of their initial unsuppressed VL and 25% (260/1028) had a follow-up VL within time and the VL result was either re-suppressed or resulted in a switch to second-line within 90 days of confirmed failure (Fig 1).

The viral load cascade improved if longer time intervals were allowed. Considering all follow-up VLs done among the 1,028 patients with unsuppressed VL during the study period, 759 (74%) had a follow-up VL during study period (median time interval to follow-up VL: 6 months (range 0–29)). Of these, 416 had virological failure. Of the 416 with virological failure, 227 (55%) were either already on second-line (n = 33) or switched during follow-up (n = 194). Not considering time intervals, 563 (55%) of the 1,028 patients with unsuppressed VL were followed as per guidelines during the study-period. Table 2 provides detailed information on outcomes of all patients with unsuppressed VL during the study period, stratified by rural clinics versus hospital.

**Table 2:** Viral load outcomes.

	All	Urban	Rural
**N individuals**	9949	4140	5806
- *N viral loads*	24,948*	11,011	13,932
- *Viral load tests per person*, *(IQR*^*$*^*) [range]*	3 (2–3) [1–9]	3 (1–3) [1–8]	2 (1–3) [1–9]
**Viral load results**			
- *Always suppressed (<20 c/ml*^†^*)*	6175 (62%)	2382 (58%)	3791 (65%)
- *≥ 1 low-level viremia (20–999 c/ml)*	2690 (27%)	1269 (31%)	1420 (24%)
- *≥ 1 elevated VL (≥ 1000 c/ml)*	1084 (11%)	489 (12%)	595 (10%)
**Patients with VL ≥1000 c/ml and at least 6 months follow-up**	*N = 1028*	*N = 463*	*N = 565*
- *Had follow up VL*	759 (74%)	362 (78%)	397 (70%)
- *Did not have follow up VL*	269 (26%)	101 (22%)	168 (30%)
**Patients with VL≥1000 c/ml and a follow up VL**	*N = 759*	*N = 362*	*N = 397*
*Median months to follow up VL*, *(IQR) [range]*	6 (4–8) [0–29]	5 (4–7) [0–29]	6 (5–9) [0–21]
- *<3 months*	66 (9%)	31 (9%)	35 (9%)
- *3–6 months*	344 (45%)	195 (54%)	149 (38%)
- *6–9 months*	200 (26%)	80 (22%)	120 (30%)
- *9–12 months*	91 (12%)	30 (8%)	61 (15%)
- *≥12 months*	58 (8%)	26 (7%)	32 (8%)
**Result of follow up VL**			
- *<1000 c/ml*	343 (45%)	201 (56%)	142 (36%)
- *≥1000 c/ml*	416 (55%)	161 (44%)	255 (64%)
**Patients with first and second VL ≥1000 c/ml:**	*N = 416*	*N = 161*	*N = 255*
- *Already on second-line ART*	33 (8%)	13 (8%)	20 (8%)
- *Had >0*.*5 log drop between first and second VL*	34 (8%)	9 (6%)	25 (10%)
- *Switched to second-line*	194 (47%)	106 (66%)	88 (35%)
- *Died*	11 (3%)	1 (1%)	10 (4%)
- *Transferred out*	6 (1%)	0	6 (2%)
- *Declared LTFU*^§^	10 (2%)	3 (2%)	7 (3%)
- *VL results came <8 weeks from data censoring*	6 (1%)	1 (1%)	5 (2%)
- *Had third VL*	62 (14%)	10 (6%)	52 (20%)
- *On-going EAC*^*£*^	20 (5%)	6 (4%)	14 (5%)
- *Ongoing poor adherence according to health care provider*	7 (2%)	2 (1%)	5 (2%)
- *Migrated to South Africa*	1 (<1%)	0	1 (<1%)
- *Other/Unknown*	32 (8%)	10 (6%)	22 (9%)
**Patients not switched after two VL ≥1000 c/ml and result of a third VL available:**	*N = 62*	*N = 10*	*N = 52*
- *<1000 c/ml*	13 (16%)	5 (50%)	8 (15%)
- *≥1000 c/ml*	49 (84%)	5 (50%)	44 (85%)
**Patients switched to second-line**	*N = 194*	*N = 106*	*N = 88*
- *Median weeks to switch (from 1*^*st*^ *VL ≥1000 c/ml)*, *(IQR) [range]*	39 (26–52) [8–121]	29 (22–45) [8–113]	47 (38–55) [10–121]
- *Median weeks to switch (from 2*^*nd*^ *VL ≥1000 c/ml)*, *(IQR) [range]*	11 (5–23) [2–95]	6 (5–13) [2–62]	17 (12–26) [2–95]
- *Switched within 90 days of second VL ≥1000 c/ml*	105 (54%)	26 (30%)	79 (75%)
**VL result after switch to second line**	*N = 142*	*N = 85*	*N = 57*
- *<1000 c/ml*	131 (92%)	79 (93%)	52 (91%)
- *≥1000 c/ml*	11 (8%)	6 (7%)	5 (9%)

* N = 5 samples missing location information

^$^ IQR = interquartile range, VL = viral load

^§^LTFU = Lost to follow up

^£^ EAC = Enhanced Adherence Counselling

^†^c/ml = copies/millilitre

### Sub-group analyses

Women were more likely to get a follow-up VL in case of unsuppressed VL (69% versus 76%, p = 0.015). In case a follow-up VL was done, prevalence of virologic failure was similar in men and women (55% versus 56%, p = 0.23).

At follow-up VL the rural clinics had higher rates of virologic failure (2 consecutive VL≥1000 copies/mL) compared to the district hospital (64% versus 44%; p<0.001). Providers in rural health facilities were less likely to switch patients with virological failure to second-line (35% versus 66%, p<0.001) and more likely to request a 3^rd^ VL instead (20% versus 6%, p<0.001). If switched to second-line, median time from confirmed virological failure to switch was longer in rural clinics (17 versus 6 weeks, p<0.001). In patients where a third VL was requested instead of switch to second-line after confirmed virologic failure, the 3^rd^ VL was again ≥1000 copies/mL in 84% of patients.

Of the 142 (65%) with a VL test after switch to second-line, 8% had an unsuppressed VL and the remaining 92% achieved re-suppression after switch. In total 9,949 patients received 24,948 VLs to trigger 194 switches to a second-line regimen (129 VLs per regimen switch).

### Turn-around time of VL results

The majority of samples from the district hospital were registered in the laboratory on the same day they were sampled compared to 8 days (IQR 1–17) in rural health facilities (Table 3). Median time from sample registration at the laboratory to processing the results was 5 days (IQR 0–12) days and did not vary by location. The turn-around time for samples was a median of 9 days (IQR 2–21) with 16% of samples taking more than 28 days from blood draw to testing (Table 3). Late turn-around time varied significantly by location (2% urban versus 27% rural, p<0.001).

**Table 3:** Sample processing times by health center location.

	All	Urban	Rural
**Total viral load results**	24,948*	11,011	13,932
- *2015 (December)*	124	123	1
- *2016*	7770	4302	3468
- *2017*	10,498	3853	6643
- *2018 (follow-up only)*	6556	2733	3820
**Registration time (blood draw until registration)**			
- *Time >2 weeks*, *n (%)*	4343 (17%)	22 (<1%)	4317 (31%)
- *Time in days (IQR*^*$*^*) [range]*	0 (0–10) [0–393]	0 (0–0) [0–58]	8 (1–17) [0–393]
**Processing time (registration until testing)**			
- *Time >2 weeks*, *n (%)*	4774 (19%)	1669 (15%)	3103 (22%)
- *Time in days (IQR*^*$*^*) [range]*	5 (0–12) [0–198]	5 (0–9) [0–198]	5 (0–14) [0–147]
**Turnaround time (blood draw until testing)**			
- *Time >28 days*, *n (%)*	4042 (16%)	261 (2%)	3778 (27%)
- *Time in days (IQR*^*$*^*) [range]*	9 (2–21) [0–393]	5 (0–9) [0–199]	15 (7–30) [0–393]

* 5 samples are missing location information

^$^ IQR = interquartile range, VL = viral load

## Discussion

In this prospective cohort study, we assessed the VL cascade after introduction of routine VL monitoring in 11 rural clinics and the district hospital in a district in Northern Lesotho in December 2015. Among those patients with a first unsuppressed VL, only 25% of individuals were managed correctly according to WHO guidelines. The remaining either did not receive a follow-up VL within 6 months (60%) or were not switched to a second-line regimen in the case of confirmed virological failure (15%). Women were more likely to have a follow-up VL done after an initial unsuppressed VL, but not more likely to have virological failure or to switch to second-line than men. In rural clinics patients had higher rates of virologic failure, providers were, however, more reluctant in switching patients to second-line and if switched, the time interval between confirmed virologic failure and switch was substantially longer than in the district hospital. On average, it took 129 VLs to trigger one switch in ART regimen.

Publications on the VL cascade after implementation of routine VL monitoring remain scarce. However, all published data indicate large gaps in the cascade. Recently Etoori and colleagues reported findings from a rural region in Swaziland where 62% of patients with unsuppressed VL received follow-up testing within 6 months and only 43% among those with confirmed virological failure switched to second-line regimen[13]. Petersen and colleagues report from cohorts in South Africa and Uganda that 180 days after confirmed virological failure, only 30% of patients were switched to second-line ART [9]. In another cohort analysis from Uganda, the cumulative incidence of switching at 6, 12, and 24 months following virological failure were 30.2%, 44.6%, and 65.0%, respectively, and not being switched was associated with higher mortality[8]. The latter two studies were conducted in specialized urban HIV centers. In our study, performance was similar to these studies. However, we found that the performance of the rural nurse-led clinics was poorer compared to the hospital: only 34% with virological failure were switched to second-line ART versus 66% at the hospital and among those switched, median time from second unsuppressed VL to switch was 17 versus 6 weeks. The successful model of task-shifting and decentralization of ART care in Lesotho [14] may be vulnerable when it comes to the management of treatment failure, which could favor the spread of resistant HIV strains and endanger past achievements [15,16].

There is no doubt that VL monitoring is the method of choice to monitor virological success of ART[2] and, in combination with adherence support and timely switch to second-line, plays a key role in preventing further emergence of drug-resistances in resource-limited settings[15,17]. However, our data indicate that in real life, cost-effectiveness of VL monitoring may be substantially lower as the majority of unsuppressed VL test results do not trigger any action, i.e. no follow-up VL testing. Médecins Sans Frontières estimated the comprehensive cost of VL monitoring to be 34 USD per test in Lesotho (USD 20 for consumables) [18]. Considering this price in our study, cost for VLs done to trigger one change of ART regimen amounted to 4,386 USD. Cost-effectiveness of VL monitoring may be even lower in remote rural clinics where performance in the VL cascade is worse.

Therefore, ART programs need to combine roll-out of VL monitoring with strategies that ensure that patients with virological failure are followed-up and that confirmed virological failure results in action, in order to achieve virological re-suppression[3,7,19,20]. Sunpath and colleagues showed in three clinics in South Africa that introduction of a “viral load champion”, a nurse who was specifically assigned to ensure correct follow-up of VLs, resulted in a higher proportion monitored according to schedule[21]. Venables et al. report that SMS alerts to clinics and patients when VL results are out was well perceived and could have the potential to help patients adopt a more active role in the self-management of their HIV disease [22]. Such strategies, however, still have to be tested at a larger scale and as part of routine care at district or national level. Recently, Shroufi and colleagues proposed a more pragmatic approach where a one-time VL≥1000 copies/mL would already trigger a switch to second-line, thus reducing delays caused by adherence counselling visits and follow-up VL. In a simulation model, this strategy reduced mortality and the incidence of AIDS related events[23]. In a systematic review, Barnabas and colleagues propose a more holistic approach that does not only focus on those with unsuppressed VL but uses VL results to differentiate care: For patients with suppressed VL, frequency of follow-up can be reduced, meanwhile freeing up capacities to manage those failing ART. This strategy may be the key to ensuring VL testing as a cost-effective strategy to monitor patients on ART[24].

This study has several limitations. First, this open cohort only includes patients who received at least one VL measurement. We do not have precise data on how many patients did not receive any VL determination. The overall percentage of patients with unsuppressed VL may thus be higher. Second, we do not have resistance testing results from patients with virological failure. We thus cannot determine if the provider’s decision to postpone switch to second-line in some patients due to suspected on-going poor adherence was justified or not. In a previous study in the same setting, in only 13% of patients with virological failure genotypic resistance testing found no major drug resistance mutation [25]. Third, the use of routine data is prone to suboptimal data completeness and quality, as well as inconsistent in confirming of patients’ status (i.e. lost to follow-up) and, hence, might bias our findings. Fourth, we do not know the exact reasons why patients were not followed according to local guidelines. Structural barriers may negatively impact on the care cascade (i.e. difficult access to health facility in rural setting for the patients, or missing laboratory supplies for the health care staff), but also provider factors such as lack of knowledge about the guidelines and the switching process or reluctance to switch. Future studies are needed to investigate the reasons.

### Conclusions

In line with previous publications, our data from Lesotho show that in a real-life setting the potential of routine VL monitoring for patients taking ART is not optimized. A lack of timely follow-up after a first unsuppressed VL as well as low switching rates among patients with confirmed virological failure, reduce the potential benefit of routine VL monitoring in resource-limited settings. Future studies should look at models of care that ensure that VL results are met with an action. Such models should not only rely on more staff training and supervision, but make use of innovative information technology approaches that–if successful–can be scaled up.
